# Brain on the stage – Spotlight on nervous system development in zebrafish: EMBO practical course, KIT, Sept. 2013

**DOI:** 10.1186/1749-8104-8-23

**Published:** 2013-12-19

**Authors:** Steffen Scholpp, Lucia Poggi, Mihaela Žigman

**Affiliations:** 1 Institute of Toxicology and Genetics (ITG), Karlsruhe Institute of Technology (KIT), Karlsruhe 76021, Germany; 2 Centre for Organismal Studies (COS), University of Heidelberg, Heidelberg 69120, Germany

**Keywords:** Development, Live imaging, Microscopy, Nervous system, Zebrafish (*Danio rerio*)

## Abstract

During the EMBO course ‘Imaging of Neural Development in Zebrafish’, held on September 9–15^th^ 2013, researchers from different backgrounds shared their latest results, ideas and practical expertise on zebrafish as a model to address open questions regarding nervous system development.

## Background

The EMBO practical course on ‘Imaging of Neural Development in Zebrafish’ took place at the Institute of Toxicology and Genetics (ITG) and the European Zebrafish Resource Center (EZRC), at the Karlsruhe Institute of Technology (KIT) in Germany, on September 9–15^th^ 2013. Here, we provide the readers who might have missed this event with a glimpse of the recent microscopy techniques and experimental approaches applied by research groups using zebrafish as a model to understand the molecular and cellular mechanisms in play during nervous system development.

### From the beginnings of cellular neuroscience to analysing the “second brain” in zebrafish

In the past, before transgenesis and fluorescent tracers in zebrafish were available, researchers had already realized the advantages of and ease of embryonic manipulations in zebrafish embryos in the study of nervous system development. Single cell transplantations had been established in order to explore, for example, intrinsic and extrinsic influences on neuronal subtype specification in the spinal chord, as featured by Judith Eisen. Nowadays, she applies modern fluorescent tagging and genetic manipulation during single cell transplantations to study the reciprocal interactions between enteric nervous system functions, the immune system, and the bacterial milieu of the intestine [1]. Live imaging of the intact gut, with its inherent peristalsis, represents a challenging new research field with an evolutionary perspective. At the course, the participants had the opportunity to troubleshoot the high precision practical skills required for single cell transplants with Judith Eisen.

### From intracellular patterning to morphogenetic tissue field patterning

Establishment of patterns either in fields of cells or within individual cells may both require morphogen signalling. It is intriguing to understand how the gradient of a pattern would either be transduced from one pole of a cell to another, from one cell to the neighbouring one, or even across an entire tissue. As an example for how single cell polarization controls tissue shape and function, the mitotic spindle is precisely polarized in 3D during symmetric neural keel mitoses, required for neural tube morphogenesis. How that could be regulated mechanistically was highlighted by Mihaela Zigman, by showing how quantitative confocal imaging, deep tissue two-photon microscopy, and fluorescent protein trapping approaches helped to reveal two regulators of mitotic spindle orientation: Scribble [2] and a novel regulator of cell polarization, a Hox gene. Paula Alexandre delved into cell polarization in asymmetrically dividing progenitor cells in the spinal cord [3]. She introduced how transient basal cellular extensions in progenitors cells, that she proposed to be actively contributing to Delta/Notch signalling between laterally adjacent neural progenitors, could be leading to a segmental pattern of organization of neurons in the spinal cord.

Finally, how could cellular patterns contribute to building up morphogen gradients? Steffen Scholpp and his team imaged Wnt8a ligand localization and found that Wnt8a is loaded on tips of actin-based filopodia, which are required for Wnt propagation, contact formation, and activation of signalling in neighbouring epiblast cells of the zebrafish embryo. Formation of those filopodia was found to be regulated by Cdc42 and are hypothesized to be required for proper anteroposterior patterning of the early central nervous system (CNS). The lecture was accompanied with a time-lapse experiment showing Wnt positive filopodia in the neural plate.

### Role for cell lineage in neurogenesis: focus on the retina

**Figure 1:**
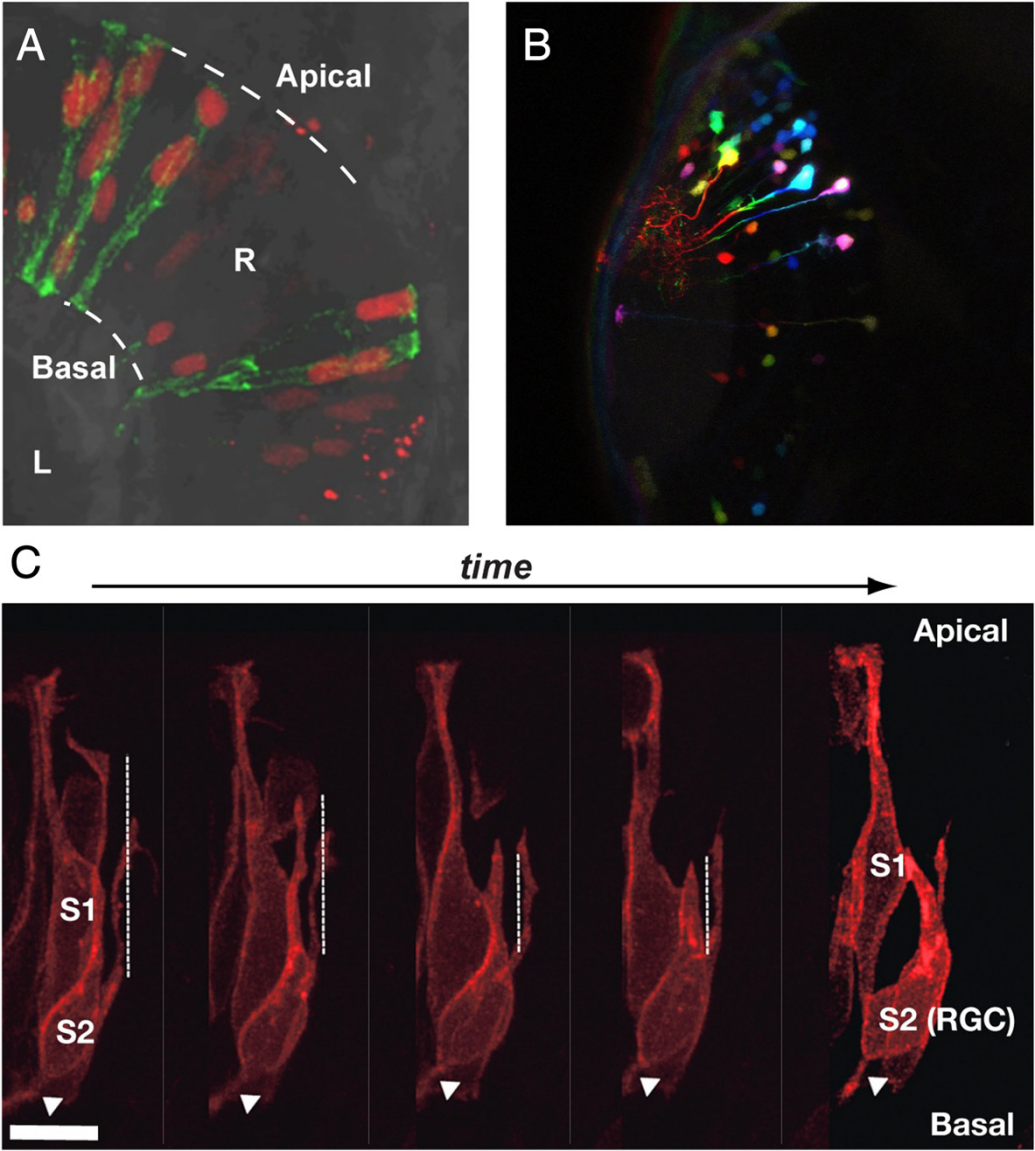
**Mosaic labelling techniques in the zebrafish embryo. (A, C)** Visualisation of individual transplanted retinal cells from donor embryos labelled with either H2B-RFP and membrane tagged-GFP **(A)** or the Atoh7-gap43RFP transgene **(C)** at 28 hpf. In **(C)** frames from a time-lapse series showing apical process retraction from the apical surface (up, see vertical dotted line) and axon extension at the basal surface (white arrow) of the s2 sibling that differentiates into a retinal ganglion cell after asymmetric division. R, Retina; L, lens. Scale bar is 10 μm. **(B)** Few small cell clones labelled by electroporation of CMV:mCitrine. Acquired images were pseudo-coloured afterwards according to z-level to visualize individual cell clones in the brain at 32 hpf. In **(A, C)**, the retina is oriented dorsal up. **(B)** Dorsal view of the midbrain. The data presented in this figure were acquired by the course participants with the help of Wiebke Sassen (Braunschweig), Shahad Albadri and Anne-Laure Duchemin (Heidelberg).

In a group of lectures, live imaging in the zebrafish retina was shown as an approach to investigate individual neural stem cell (NSC) behaviour in the developing and mature vertebrate CNS. Bill Harris discussed how direct observation of full clone formation and neural fate acquisition provides essential information challenging common models of neural fate acquisition *in vivo*. In light of this, efforts towards reconstruction of the entire retinal lineage from individual equipotent retinal progenitors to generate a map of vertebrate retina *in vivo* were made, and the relative importance of stochastic versus deterministic influences on proliferation and cell fate acquisition within individual cell lineages were readdressed by his team [4]. A quadruple transgenic line marking four different retinal cell types with different fluorophores, a “polychrome retina”, is an exciting new tool that opens up the way to further improvements in simultaneous imaging of different neural lineages. Submerging into neuronal diversity, Lucia Poggi discussed how asymmetric cell division and occurrence of genetic programs in sibling cells might contribute to the final cellular subtype composition of retinal circuits. Focusing on amacrine and ganglion cell subtype lineages, Poggi presented the use of a combination of *in vivo* imaging of sibling cell fate (Figure 1A), cell specific inducible systems, and functional approaches to address relationships between genetic programs and respective cell division patterns [5], on which the participants had an opportunity to practise (Figure 1A,C).

Exploring cell lineage commitment post-embryonically, Jochen Wittbrodt tackled long-term individual cell tracking of neural stem cells (NSC) located in the ciliary margin of the medaka fish retina, investigating the potency of NSCs. His team recently unveiled that neural retina and retinal-pigmented epithelium are both maintained by NSC-independent populations of fate-restricted NSCs [6]. They have now gone a step further and developed a technique (termed “Gaudi”) where light sheet microscopy is combined with genetically encoded fluorescent lineage tracing to specifically mark individual post-embryonic NSCs and the fate of their progeny in space and time.

### Let’s get connected: synapses, neurons and neural circuits

Following the establishment of different cell types, neuronal circuit formation is inherent for proper retinal function. Stephan Neuhauss talked about optokinetic response in zebrafish and functional approaches to study glutamate signalling at the photoreceptor-to-bipolar synapse [7]. Depending on the bipolar cell type, glutamate either depolarizes (activates) or hyperpolarizes (inhibits) the postsynaptic cell. The evolutionary perspective of the role of glutamate transporters in this synaptic transmission was presented.

Moving to the brain, Reinhard Köster presented the elegant use of non-invasive *in vivo* mapping of cerebellar neuronal connectivity by trans-neuronal tracers to identify the efferent network of Purkinje cells (PC). In addition, his team revealed, by imaging of calcium-activity in PCs during stereotypic behaviour of zebrafish larvae, that different PC territories are involved in regulating different behaviours such as saccadic eye movements or swimming. This was supported by optogenetic analysis in which PCs were transiently inhibited during behaviour performance showing that indeed the PC layer is subdivided into different functional domains. Johann Bollmann introduced the role of synaptic connections in biological information processing using a set of *in vivo* methods. He makes use of high-speed functional imaging of swim patterns and electrophysiology combined with a virtual reality environment to obtain complex stimulus-response relationships [8]. Analysing visual information processing he unveils how visual input and sensory feedback controls guided motor behaviours. Further application of electron microscopic techniques will be important in the future to add ultrastructural information on synaptic connectivity.

Following up on this line, Martin Meyer showed how modern light microscopy can be used to functionally study retinal ganglion cells, the output neurons of the retina, and the mechanisms of how this information is processed and utilised by local circuits within the brain [9]. Conversation between the eye and the brain changes as visual circuits mature in complexity. The elegant use of GAL4/UAS-driven synaptophysin calcium indicator along with two-photon functional imaging [10] was practised on by course participants.

Matthias Carl moved into habenular neural circuit development, showing the application of long-term imaging to study communications between left and right brain hemispheres during axon extension. By tracking the entire network in combination with focal laser cell ablation, his team revealed that habenular axon elongation is coordinated by a second neural network in the thalamus, facilitating the exchange of information between the two sides of the brain. Consistently, unilateral interference with thalamic network formation results in the bilateral arrest of habenula axon extension.

Florian Engert gave a thrilling and involving talk addressing the mechanisms necessary for zebrafish larvae to learn by developing an operant learning paradigm. The use of an infrared laser beam towards a head-fixed animal with concomitant tail-motion recordings and online analysis with a high-speed camera was used in a closed loop system to control the power of the laser. Intriguingly, zebrafish are found to learn quickly and that they can re-learn a reversed paradigm. Development of such unique novel screening systems provides a possibility to address the requirement of individual neuronal classes for specific types of learning behaviour.

The sessions on general principles governing the assembly of neural circuits *in vivo* highlighted the importance of the zebrafish model in the field [11] and revealed new challenges for future research when combining functional imaging, genetics, and behavioural studies.

### Removal of dead neurons and nervous system regeneration

The capacity of zebrafish to regenerate nervous system tissue functionally has more recently lead to the use of zebrafish as a valuable model for adult vertebrate regeneration [12]. Underlying the mechanisms of recognition and removal of sick and injured neurons in the brain, microglia play a pivotal role [13]. To understand the control of microglial activity, Francesca Peri and her group takes advantage of live imaging in the intact zebrafish embryonic brain. The use of forward and reverse genetic approaches with quantitative imaging to tackle the mechanisms of microglia attraction and action were discussed. Moreover, hands-on and technical troubleshooting sessions on fluorophore photoconversion to track microglia and genetically encoded ablation systems to kill neurons and to experimentally study removal of apoptotic cells, provided participants with practical training.

The mechanisms behind the regeneration of the adult zebrafish brain are widely unknown. In search for underlying signalling pathways, Uwe Strähle reported on mapping the expression of more than 1,000 transcriptional regulator genes in the adult zebrafish telencephalon upon stab injury [14] and found expression changes of a subset of these factors in regeneration upon large injuries in the telencephalon. A negative feedback loop that limits the proliferative response of the injured brain was discussed.

### At the frontiers of light microscopy

Fundamental principles and the advantages of different modern microscopy techniques were introduced by Jan-Felix Evers. Although the newest advances in light microscopy promise to push detection sensitivity and image resolution to ever-new boundaries, many of them are not yet applicable for imaging developmental processes. Uli Nienhaus presented the fundamentals of optical nanoscopy, including photo-activated localization microscopy (PALM/dSTORM) and STED/RESOLFT microscopy techniques as well as the analysis of fast dynamics such as molecular diffusion in cultured cells by using fluorescence correlation spectroscopy and raster image correlation spectroscopy [15]. Martin Bastmeyer provided direct comparisons between structural illumination microscopy (SIM) and PALM/dSTORM and discussed how live-cell imaging using SIM could become standard routine lab work.

**Figure 2:**
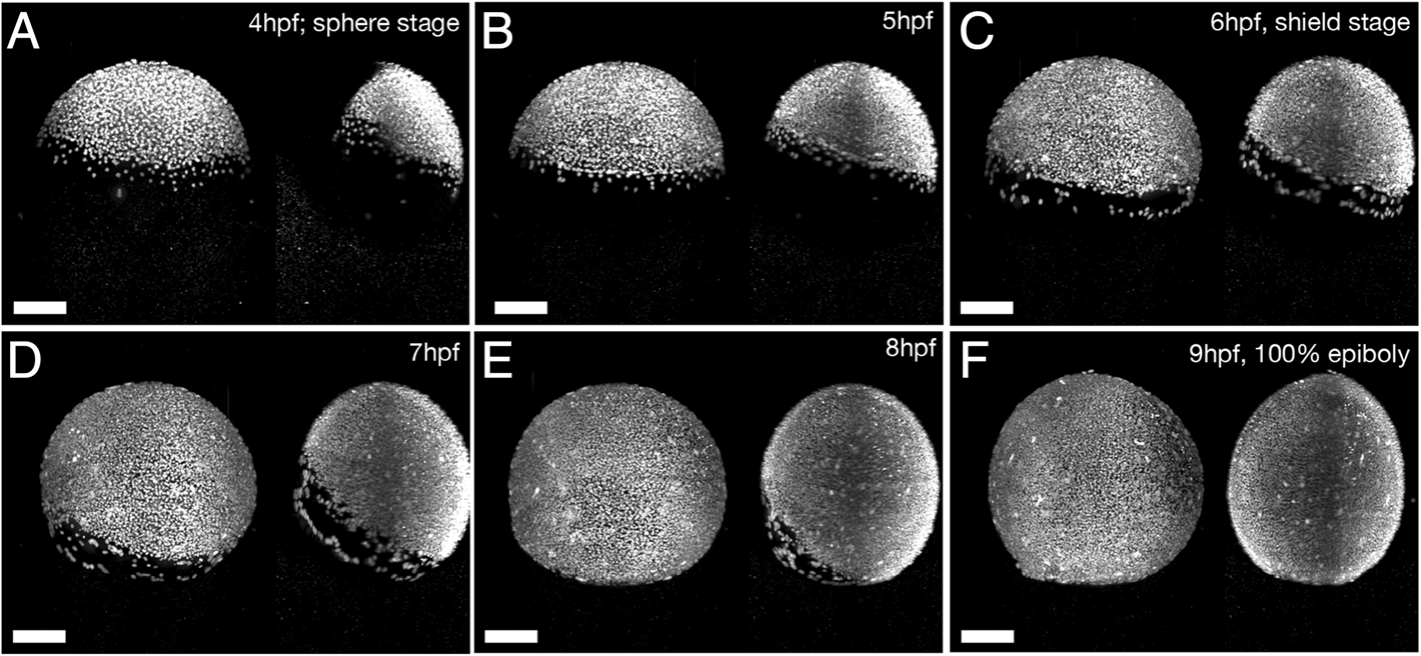
**Imaging of zebrafish gastrulation by digital light sheet microscopy.** Maximum-intensity projections of H2B-GFP labelled wild type zebrafish embryo at the indicated times and subsequent developmental stages from **A** to **F**. The data presented in this figure were acquired by the course participants with the help of Andrey Kobitski, KIT.

Although the most recent developments in high-resolution imaging and optical nanoscopy are very exciting their application to study the zebrafish nervous system *in vivo* are still challenging. The possible impact of digital scanned laser light-sheet fluorescence microscopy, especially when combined with routine image analysis tools, proved to be very exciting for the course participants, who noticed that the system had already evolved to a very user friendly version as shown in Figure 2.

### Analysis and mapping of acquired imaging measurements

Quantitative image analysis is essential for the assessment of microscopic measurements. The use of freely available and user upgradable microscopy data analysis software (Fiji/ImageJ) was practically demonstrated by Carlo Beretta during the course. Matthias Carl showed how development of the large habenula neuronal network could be followed for more than four days using optimized long-term high-resolution two-photon microscopy. Moreover, developing macros and plugins for Fiji, they were able to process hundreds of gigabytes of time-lapse data within a couple of hours [16], to visualize neuronal network formation on a single axon level in a 3D volume. Such tools are crucial for identification of key steps during neural circuit development.

As the ultimate goal is direct visualization and quantification of the biological processes in four dimensions, software programs specialized for 3D image analyses are indispensable. Stefan Terjung provided guidance for the use of such image analysis tools and presented Imaris software (Bitplane). Alessio Paolini, Shahad Albadri and Anne-Laure Duchemin introduced features from the Volocity software (Perkin Elmer) with which the participants experienced 3D tracking and image rendering on imaging data acquired earlier at the course.

An anatomical atlas for 3D high-resolution spatial comparison of gene expression in the larval zebrafish brain called ViBE-Z [17] was presented by Wolfgang Driever. The application is based on a freely available web interphase that automatically maps gene expression data with cellular resolution to a 3D standard larval zebrafish brain combining quality enhancements of the data, use of automatically detected anatomical landmarks, and annotations of all major brain regions. Efforts to add more stages and development of additional anatomical reference systems were discussed.

### Upcoming methods for loss of function in zebrafish

Recent advances in genome editing in zebrafish were highlighted by Bettina Schmid. Comparison of Zinc finger nucleases, transcription activator-like effector nucleases(TALENs), and the recently established CRISPR/Cas9 system were introduced and the effective use of this system to knock-out genes with high specificity [18], as well as a successful knock-in example, were demonstrated, both of which are important for the future development of zebrafish research.

### European Zebrafish Resource Center (EZRC) and Screening Center at ITG/KIT

In 2012, KIT established the EZRC, which keeps zebrafish lines from European researchers and ships them worldwide, offers sequencing and screening services, and operates the local experimental fish facility. For the course, Robert Geisler introduced the EZRC, provided us with zebrafish embryos and injection facilities, and the basics about zebrafish husbandry.

Ravindra Peravali introduced the Screening Center at KIT, showed new techniques for high-resolution screening using an automated microscopy platform [19], combined with methods to systematically orient zebrafish embryos and detect regions-of-interest. These techniques were complemented by quantitative behavioural analysis screening system and an automated image analysis software, developed at the ITG.

### What we’ve done and where to go

“Time-lapse imaging of zebrafish development could hardly be simpler”, as Francesca Peri said, yet, new techniques need to be developed and existing ones refined. The use of zebrafish as a nowadays-irreplaceable model to dissect open questions in vertebrate development is based on the unique transparency of the zebrafish embryo making it ideal for live imaging, with the added advantage of the ease of embryonic manipulations and more recently developed methods of functional imaging and laser manipulations, as well as possibility of genetic manipulations. In a series of experiments, course participants explored ways to mark individual cells using either cell transplants, fluorophore photo-conversion, or electroporation of individual cells (Figure 1). These were used to follow-up on cell lineage confinement over time in 3D embryos and were complemented by direct assessments of neuronal activity in individual neurons of living larvae.

The forum of this course, with participants from diverse backgrounds, further underscored the need for higher resolution, speed and sensitivity in new light microscopes, importantly applicable to models in developmental biology. Despite the optical advantages in the zebrafish, many recent techniques still have not bridged the gap between imaging cultured cells grown on a slide to deep tissue live imaging. Furthermore, the latest imaging technologies are not at the stage of being routinely applicable for developmental biologists, therefore urging for close collaboration between biologist and physicist. Apart from the optical technology, development of software able to deal with large data sets easily will be crucial. Also, the course made it evident that many research centres, at least in Europe, count with the latest commercial models of light microscopes, whereas training and advice of early career scientist on what system to use to best tackle the questions they are wishing to investigate is often lacking. Although the use of zebrafish has already proven to greatly contribute to our understanding of vertebrate nervous system development, new advances in genome editing together with functional live imaging and optical probing will pave the way of future research in the field. Imaging of zebrafish development could thereby serve as a springboard with possible implications to translational research.

### Consent statement

Written informed consent was obtained from the researchers involved in obtaining the results in Figure 1 and Figure 2 of this manuscript.

## Abbreviations

CNS: Central nervous system; EZRC: European Zebrafish Resource Center; ITG: Institute of Toxicology and Genetics; KIT: Karlsruhe Institute of Technology; NSC: Neural stem cell; PALM: Photo-activated localization microscopy; PC: Purkinje cells; RESOLFT: Reversible saturable optical fluorescence transitions; SIM: Structural illumination microscopy; STED: Stimulated emission depletion; STORM: Stochastic optical reconstruction microscopy.

## Competing interests

The authors declare that there are no financial competing interests.

## Authors’ contributions

MZ conceived the report outline and wrote the manuscript; SS and LP were involved in drafting and revising it. All authors read and approved the final manuscript.
